# Effects of Nalbuphine on Gastrointestinal Function in Post-Operative Critical Ill Patients Admitted to the ICU: A Multicenter Randomized Controlled Trial

**DOI:** 10.3389/fmed.2022.836872

**Published:** 2022-02-16

**Authors:** Yun Yan, Chong Lei, Binxiao Su, Enxia Dong, Guangming Wang, Bin Li, Xinyu Li, Aiguang Li, Guifen Gan, Yu Chen, Xijing Zhang

**Affiliations:** ^1^Department of Intensive Care Unit, Xijing Hospital, The Fourth Military Medical University, Xi'an, China; ^2^Department of Anaesthesiology and Perioperative Medicine, Xijing Hospital, The Fourth Military Medical University, Xi'an, China; ^3^Department of Intensive Care Unit, The Affiliated Honghui Hospital of Xi'an Jiaotong University, Xi'an, China; ^4^Department of Intensive Care Unit, Hospital of NORINCO GROUP, Xi'an, China; ^5^Department of Intensive Care Unit, The First Hospital of Lanzhou University, Lanzhou, China; ^6^Department of Intensive Care Unit, The Second Affiliated Hospital of Xi'an Medical University, Xi'an, China; ^7^Department of Intensive Care Unit, Aerospace General Hospital, Xi'an, China; ^8^Department of Intensive Care Unit, Qinghai University Affiliated Hospital, Xining, China

**Keywords:** nalbuphine, fentanyl, opioids, GI function, intensive care medicine

## Abstract

**Background:**

Gastrointestinal (GI) function can be a significant problem in critically ill patients and is associated with detrimental outcomes. The administration of opioids for pain reduction is thought to contribute to GI dysfunction. We tested whether nalbuphine, a mixed agonist/antagonist opioid modulator, can promote GI recovery in postoperative critical patients admitted to the intensive care unit (ICU) and compared it with fentanyl, a selective mu opioid receptor (MOR) agonist.

**Methods:**

This is a multicenter, single-blind, randomized controlled trial to investigate whether nalbuphine improves the GI recovery in ICU patients after surgery, and compared it with fentanyl. The primary outcome was the time to first defecation. Secondary outcomes included the use of sedatives, enemas or laxatives, the acute gastrointestinal injury (AGI) grade, the incidence of vomiting, and the lengths of ICU and hospital stays.

**Results:**

We randomized 436 patients, and a total of 369 patients were included in the modified intention-to-treat population (mITT) (185 to the nalbuphine group and 184 to the fentanyl group). The baseline demographic characteristics of the two groups were comparable after randomization. There was no significant difference in the time to defecation between the two groups [hazard ratio (*HR*) 0.94, 95% *CI* 0.74–1.19, *p* = 0.62]. There was no significant difference in the secondary outcomes between the two groups.

**Conclusion:**

We found no evidence that nalbuphine administration can improve the GI function in postoperative critical patients admitted to the ICU compared with fentanyl. However, the *CI* was wide and we could not exclude the clinically important difference.

## Introduction

Gastrointestinal (GI) dysfunction frequently occurs in critically ill patients; it has an incidence of 50% on the 1st day of admission to the intensive care unit (ICU); and GI dysfunction is associated with increased mortality, the length of ICU stay, and medical costs (1, 2). Opioids are important medications that affect the GI function by inhibiting GI transit, secretion, and absorption (3, 4). The mu opioid receptor (MOR) mainly mediates the inhibition of GI tract. However, opioid administration is the mainstay for pain management in patients in the ICU, as pain is common in patients who are in the ICU (5). Therefore, it is crucial to balance GI function and opioid administration. Many properties should be considered when choosing opioids.

Fentanyl is the most commonly used opioid in the ICU; it is a selective MOR agonist and has a significant role in inhibiting the GI function (6). Nalbuphine is a kappa opioid receptor (KOR) agonist and a MOR antagonist opioid drug, and its beneficial effect on GI function has been demonstrated in animal models (7, 8). However, the clinical evidence regarding its impact on GI function is limited, and the results have been contradictory (9–11). In these studies, nalbuphine was administered as a preoperative medication, but the sample size of the study was small. Data on the impact of nalbuphine on GI function in critically ill patients are limited. Therefore, in this study, we investigated the effects of the agonist-antagonist analgesic nalbuphine and the pure MOR agonist fentanyl on the GI function in ICU patients after surgery.

## Methods

### Study Design

This study was a multicenter, single-blinded, randomized, controlled trial comparing the two drugs, nalbuphine and fentanyl, in surgical patients admitted to the ICU. This study was approved by the ethics committee of Xijing Hospital (No. KY20192123-F-1). The study was registered at www.chictr.org.cn (ChiCTR1900025096).

### Study Population

The trial was conducted in 7 ICUs in China. We included patients after surgery admitted to the ICU with a critical pain observation tool (CPOT) score ≥ 3 (12). The length of the ICU stay in the patients we included was predicted to be at least 48 h. Patients were excluded if they had a high Acute Physiologic Chronic Health Evaluation II (APACHE II) score ≥ 23 (13), had an acute gastrointestinal injury (AGI) grade ≥ 3, had severe liver dysfunction (Child-Pugh Grade C), were pregnant, had long time usage of opioids and sedatives, were currently in another trial, and had contraindications for taking the study drugs.

### Study Intervention

Patients were randomized to either nalbuphine or fentanyl on a 1:1 basis. The central randomization system processed the randomization with a randomized competitive enrollment mode. We did not require the enrollment of each hospital; they can recruit until the total number of participants up to our sample size of 436. Each participating hospital can get the sequence number and the results of group randomization by inputting the simple information of patients. Our study was single-blinded; only patients did not know the randomization result because we have strict rules and regulations for opioid management. The assessors were not blinded to allocation. Nurses recorded the occurrence of vomit and the time of defecation in the ICU. After discharge from the ICU, we followed the time of defecation and secondary outcomes once a day before discharge from the hospital. We considered the potency ratio between the fentanyl and nalbuphine to be 100:l. Nalbuphine had similar analgesic effects with morphine, while fentanyl is 100 times potent compared with morphine (14, 15). The treatment group received nalbuphine, the loading dose of nalbuphine was 0.1 mg/kg, and the maintenance dose was 0.06 mg/kg/h. The control group received fentanyl with a loading dose 1 μg/kg and a maintenance dose 0.6 μg/kg/h. Clinicians could change the rate of the infusion pump to meet clinical needs of the patient, the maximum rate of pump was 4 ml/h in both groups. Both these drugs were diluted in 40 ml of 0.9% saline. The clinicians decided the duration of study drug administration that patients needed by the pain score of the patients. The clinicians decided to prescribe opioids according to clinical requirements, and the default prescription was determined by the results of randomization. The sedative drugs that were used included propofol, midazolam, and dexmedetomidine. Enemas and GI motility drugs were administered to the patients as needed. If the patients had no serious abdominal injuries, enteral nutrition could be initiated within 24 h. The variables measured during the study included the diagnosis at admission, age, lactate level, blood pressure, potassium concentration, the use of GI motility drugs, the method of nutrition, enema treatment, vasoactive drugs, and sedatives. We recorded these parameters during the first 48 h of admission to the ICU. The APACHE II score was recorded during the first 48 h of admission to the ICU.

### Outcomes

The primary outcome was the time to first defecation. Secondary outcomes were the incidence of vomit, the use of sedatives and GI motility drugs, AGI grade, the length of ICU stay, and the number of hospital days. AGI had four grades of severity to describe the GI injuries (16). The AGI I grade in our study was composed of patients without an AGI and patients with an AGI grade I.

### Statistical Analysis

For the sample size calculations, the average time to defecation in the ICU was 115.2 h with a SD of 98.4 h (17). With an alpha of 0.05 and power of 80%, a total of 360 patients needed to be recruited for a difference of 25% between the time to the first defecation. Target recruitment was set at 436 patients to account for a 20% loss to follow-up (218 in each group). We had no interim analysis.

The primary outcome was performed on the modified intention-to-treat population (mITT) basis, pre-protocol, and as-treated population. The primary analysis was assessed in the mITT. The per-protocol population excluded the patients who received the opposite drugs of randomization or who received either both or neither nalbuphine and fentanyl and the patients who had a length of ICU stay <2 days. The as-treated analysis was grouped according to the study drugs that the patients actually received after excluding the patients who received both or neither of the study drugs. The sensitivity analysis was conducted in the per-protocol and as-treated populations.

We used the R package to assess the distributions of the variables. Categorical variables were described as numbers and percentages, and continuous variables were described as the mean ± SD or medians and their 25th and 75th percentiles by different distributions. We used a Cox regression model and Mann–Whitney *U*-test in the time to first defecation. The Cox regression analysis was adjusted for age, sex, the use of sedatives, APACHE II score, and operation sites (*post-hoc* analysis). Proportional hazards assumption was verified between the treatment group and time in the model. The competing risk analysis was not required for our short observation period. The secondary outcomes were compared with Pearson's chi-squared and Mann–Whitney *U*-test. Risk ratio or median difference were calculated in the outcomes. A pre-specified subgroup analysis of the primary outcome was conducted on the following variables: age, the use of sedative and vasoactive drugs, laxatives, enemas, nutritional type, mechanical ventilation, surgical site, and ICU sites. The subgroup analyses were displayed as a forest plot with hazard ratio (*HR*) and 95% *CI*. Significance was determined by the value of *p* to investigate the effect of each variable on the defecation time of the two groups (*post-hoc* analysis). In the subgroup of ICU sites, we integrated the ICU sites with a small number of patients enrolled in performing the subgroup analysis due to the large difference in the number of enrolled patients in the different ICU sites.

Missing data were censored in the analysis of the primary outcome at the discharge from the hospital. In the secondary outcome, we had no missing data, because the secondary outcome we analyzed was within 48 h; for patients who had only 1 day in the ICU, we followed the occurrence of secondary outcomes the next day. In the exploratory analysis, we also explored the predictors of defecation in the ICU patients within 48 h by the univariate and multivariable regression analyses. We included factors based on clinical knowledge in the univariate analysis. In addition, statistically significant factors in the univariate analysis (≤ 0.05) were included in the multivariable analysis (18). The results were expressed by the odds ratio (*OR*) and their 95% *CI*. Statistical analyses were performed using R software (version 4.0.2) and SPSS software. A *p* ≤ 0.05 was considered statistically significant, and a two-sided hypothesis test was used in the analysis.

## Results

**Figure 1:**
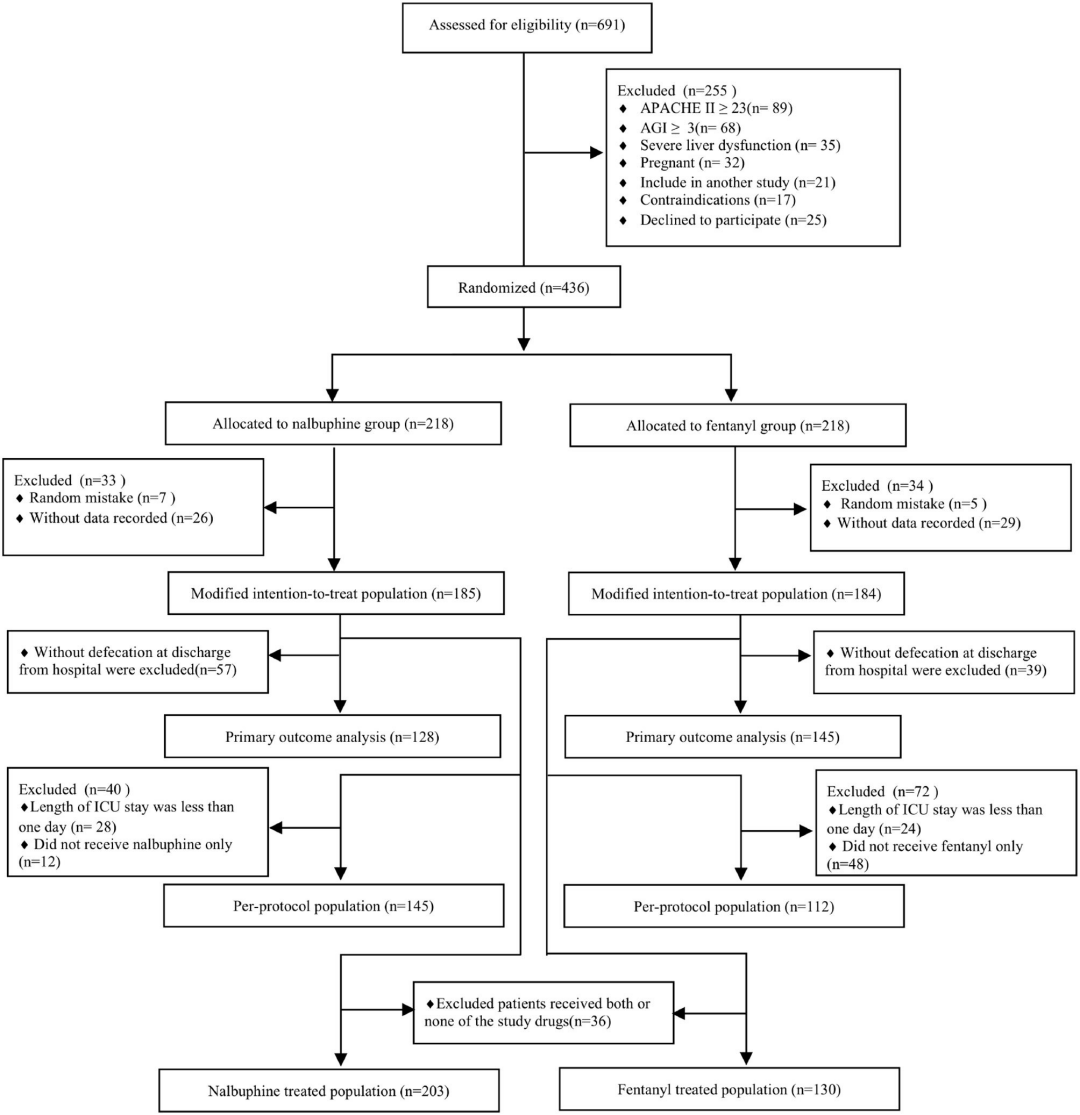
Study flowchart. ICU, intensive care unit; APACHE II, Acute Physiologic Chronic Health Evaluation II; AGI, gastrointestinal injury.

We randomized 436 patients, 218 patients were assigned to receive nalbuphine, and 218 to receive fentanyl. In all randomized patients, 369 were analyzed in the mITT (185 in the nalbuphine group and 184 in the fentanyl group). A total of 278 patients were included in the per-protocol analysis, and 333 patients were included in the as-treated analysis. We excluded patients without any recorded information and randomized number produced by randomized mistakes in the mITT analysis. The diagram shows the inclusion process of the participants in the trial (Figure 1). Recruitment was started from February 2020 to August 2021. There was no difference in the baseline characteristics between the two groups (Table 1). The diagnosis at admission and the surgical sites were equally distributed in both groups. The APACHE II score and the baseline AGI grade were well-balanced between the two groups. The patient characteristics in the per-protocol analysis and the as-treated analysis are shown in Supplementary Tables 1, 2.

**Table 1:** Demographic characteristics in the mITT population.

**Characteristics**	**Nalbuphine (*n* = 185)**	**Fentanyl (*n* = 184)**
Age, median [IOR], years	53 [41, 64]	52 [41, 64]
**Sex n (%)**		
Male	105 (56.8)	114 (62)
Female	80 (43.2)	70 (38)
Body mass index median [IQR], kg/m^2^	23.66 [21.97, 25.39]	23.44 [21.48, 25.08]
APACHE.II median [IQR]	9 [6, 11]	9 [6, 12]
**Diagnosis on admission**		
Trauma, n (%)	100 (54.1)	98 (53.3)
Spinal disease, n (%)	30 (16.2)	25 (13.6)
Digestive disease, n (%)	10 (5.4)	15 (8.2)
Other, n (%)	45 (24.3)	46 (25)
**AGI grade**		
I, n (%)	162 (87.6)	148 (80.4)
II, n (%)	23 (12.4)	36 (19.6)
Lactate median [IQR], mmol/L	1.6 [1.2, 2.1]	1.65 [1.1, 2.3]
K^+^ median [IQR], mmol/L	4 [3.7, 4.3]	4.06 [3.7, 4.31]
**Surgical site**		
Abdomen, n (%)	30 (16.2)	34 (18.5)
Limbs, n (%)	54 (29.2)	50 (27.2)
Cervical, n (%)	55 (29.7)	50 (27.2)
Other, n (%)	46 (24.9)	51 (27.7)
Systolic pressure median [IQR], mmHg	127 [112, 140]	125 [114, 139]
Diastolic pressure median [IQR], mmHg	75 [65, 81]	74.50 [64, 82]

*mITT, modified intention-to-treat population; AGI, gastrointestinal injury*.

### Primary Endpoint

**Figure 2:**
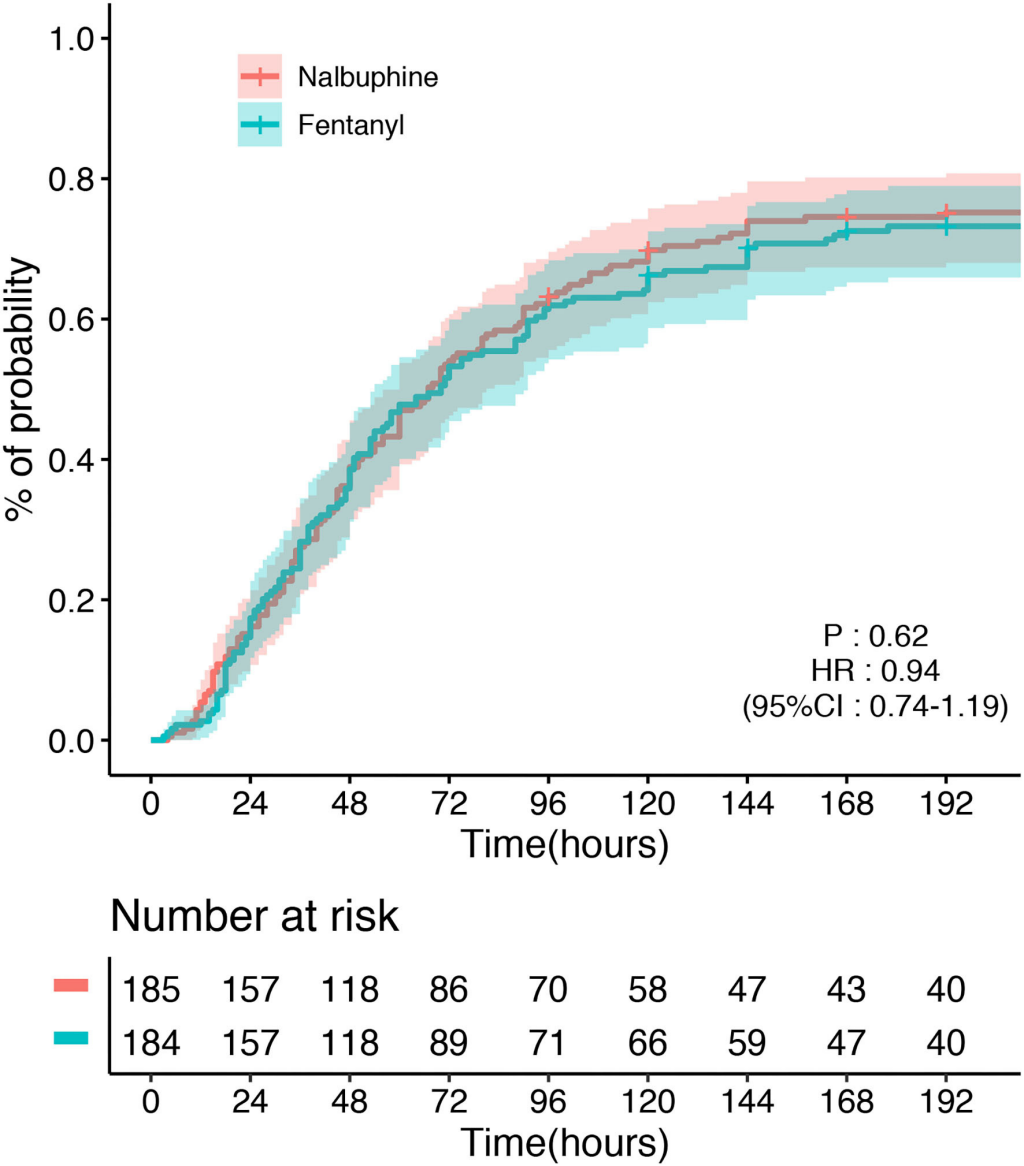
Time to the first defecation between the nalbuphine and fentanyl groups in the modified intention-to-treat population (mITT).

The primary endpoint, the time to first defecation, was not different between the two groups. The defecation occurred in 128/185 (69.2%) patients in the nalbuphine group and 145/184 (78.8%) participants in the fentanyl group. The median time of defecation in the nalbuphine and fentanyl groups was both 48 h in our study. Kaplan–Meier curves showing the time to first defecation in the nalbuphine and fentanyl groups are presented in Figure 2. The Cox regression analysis showed no significant difference between the two groups (*HR* 0.94, 95% *CI* 0.74–1.19). After adjusting the age, sex, the use of sedatives, APACHE II score, and operation sites, our study found no difference between the two groups (*HR* 0.98, 95% *CI* 0.78–1.26). The primary outcome was not different in the per-protocol population (*HR* 1.17, 95% *CI* 0.89–1.54) and the as-treated population (*HR* 1.23, 95% *CI* 0.96–1.56). There was no difference in the doses of opioids used between the two groups. The per-protocol analysis and the as-treated analysis results are shown in Supplementary Tables 1, 2. The proportional hazards assumption was valid in all the Cox models. In the mITT population, an estimated 3% of patients received fentanyl in the nalbuphine group, and 18% received nalbuphine in the fentanyl group. The exposure of the patients to the study treatment by sequence and treatment period is shown in Supplementary Figure 3. About 17.3% of the patients received other opioids in the nalbuphine group, and 12.5% in the fentanyl group. The other opioids that were frequently administered included sufentanil and remifentanil.

### Secondary Endpoints

There was no difference between the groups about the incidence of vomiting and AGI grade. Additionally, there were no differences between the groups in the use of sedatives, laxatives, and enemas. The length of ICU stay and hospital stay had no difference in both groups. The data pertaining to the secondary outcomes are presented in Table 2. The secondary outcomes in the per-protocol and as-treated analyses are shown in Supplementary Tables 3, 4.

**Table 2:** The outcomes in the mITT population.

**Outcomes**	**Nalbuphine (*n* = 185)**	**Fentanyl (*n* = 184)**	***P*-value**	**Risk ratio or median difference (95% CI)**
**AGI Grade**			0.98^a^	1.01 (0.59–1.71)^*^
I, n (%)	152 (82.2)	151 (82.1)		1.00 (0.91–1.01)^*^
II, n (%)	33 (17.8)	33 (17.9)		0.96 (0.64–1.54)^*^
Vomit, n (%)	30 (16.2)	26 (14.1)	0.54^a^	0.86 (0.53–1.39)^*^
**Sedation**			0.13^a^	
Propofol, n (%)	14 (7.6)	22 (12)		
Dexmedetomidine, n (%)	120 (64.9)	98 (53.3)		
Midazolam, n (%)	10 (5.4)	15 (8.2)		
Enema/Laxative, n (%)	46 (24.9)	47 (25.5)	0.88^a^	0.97 (0.68–1.38^*^
Length of ICU stay, median [IQR], days	3 [2, 4]	3 [2, 4]	0.8^b^	0^**^
Hospital stay, median [IQR], days	12 [8, 17]	11.5 [7, 16]	0.29 ^b^	1 (-1, 2)^**^

*AGI, gastrointestinal injury*.

a *Pearson's chi-squared;*

b *Mann–Whitney U-test;*

* *risk ratio;*

** *median difference*.

### Exploratory Analysis

**Figure 3:**
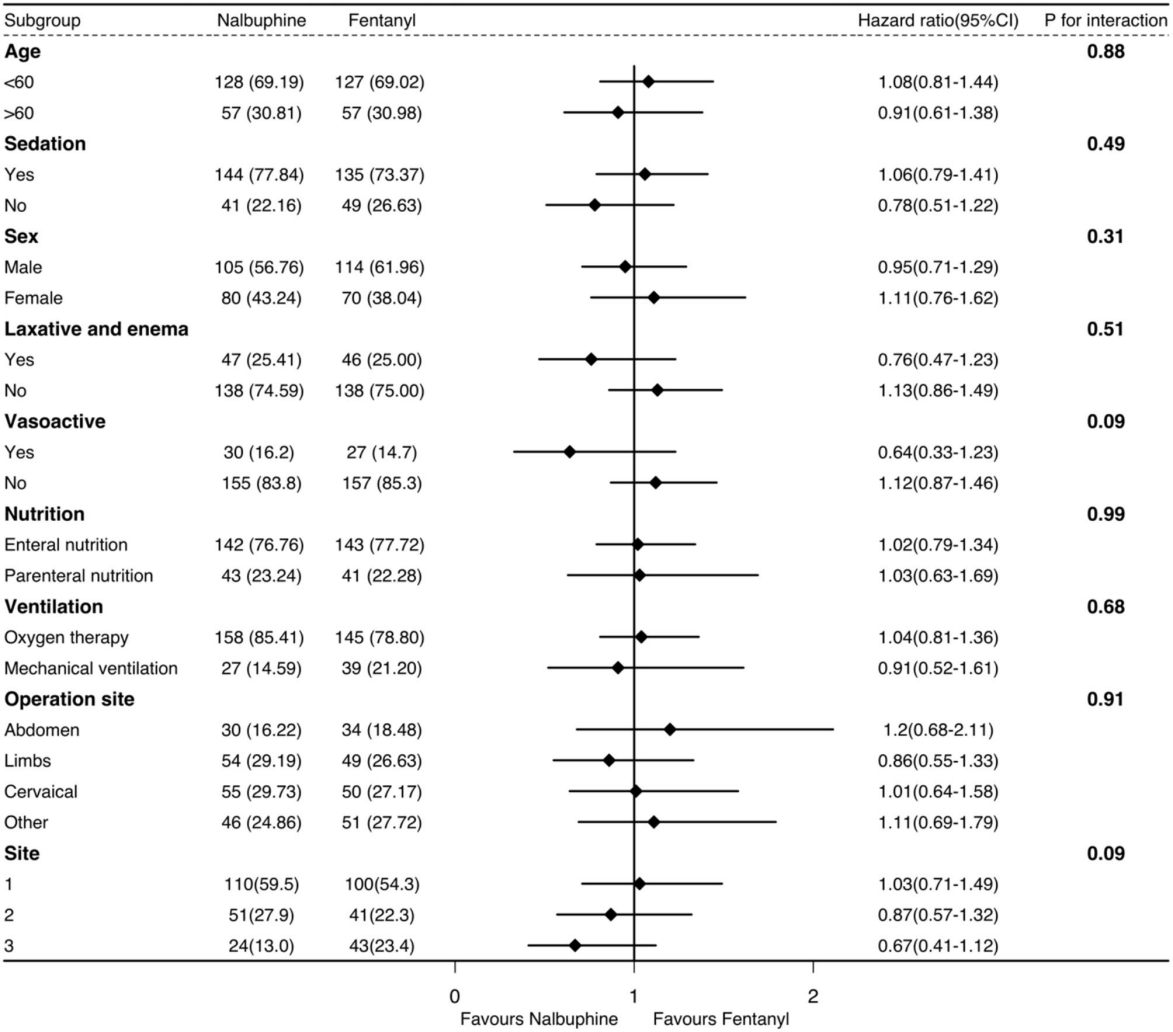
Subgroup analyses of the primary outcome in the mITT.

The univariate logistic regression analysis and multivariable model based on the variables selected in the univariate analysis indicated that the surgical site, sedatives, and the APACHE II score were associated with the long defecation time within 48 h in the ICU (Supplementary Table 5). There was significantly no difference in the subgroup analysis (Figure 3). The recruitment numbers by the ICU site and the treatment group in the mITT analysis are shown in Supplementary Table 6.

## Discussion

In this randomized clinical trial, we found no statistically significant difference in the time to first defecation in patients after surgery in the ICU in the mITT population. There was also no significant difference in the per-protocol population and the as-treated population.

In our study, 26% of the patients had no stool before discharge from the hospital. The time of defecation in our study was earlier than previous studies that reported 4 and 6 days (19, 20). We think two reasons may be associated with this. First, the illness severity of the patients we included was relatively mild to moderate, and illness severity is an important factor that impacts the GI transit by reducing the interstitial cells of Cajal in the colon (21). The patients we included had a lower APACHE II score. Second, the proportion of constipation in medical patients was higher than in surgical patients in the ICU. The reported difference in constipation incidence ranged from 7 to 36% (2, 22). About 75% of the patients we included underwent orthopedic surgery without severe GI dysfunction, and 35% of the orthopedic surgery were cervical spinal cord injuries. We usually treated these patients with regular laxatives and enemas to enhance the GI recovery. The pain intensity in ICU medical patients was significantly higher than in surgical trauma patients (23).

Opioid receptors are widely distributed in the GI tract of humans, and the different receptors are located in different areas and mediate different functions of the GI tract (24). The clinically relevant actions of opioids are mediated predominately by MOR. However, our study did not find a benefit of nalbuphine on the GI function compared with fentanyl in ICU patients after surgery. The limitations in our study may cover the real outcome. However, recent studies suggested that nalbuphine did not shorten the time to the first flatus or the first defecation compared with sufentanil in patients after laparoscopic surgery for gynecological malignancies (25). Moreover, nalbuphine did not produce a worthwhile improvement in gastric emptying compared with pethidine in patients following the laparoscopic sterilization (26). So, the effect of nalbuphine in the GI function may not be as we imagined. Maybe another partly MOR that was not antagonized or the activation of KOR that did not find in the human GI tract had an important role in the effect of nalbuphine. Therefore, the substitution with an agonist-antagonist analgesic, such as nalbuphine for a pure MOR agonist in the ICU needs to consider more. GI dysfunction in critically ill patients is insufficiently understood (27). The different effects of the agonist-antagonist analgesic and pure MOR agonist on the GI function require further research.

We had large amounts of patients excluded in the mITT analysis. The randomized mistakes have occurred in the provider who did not participate in our study, but they know the randomization process. Meanwhile, for patients excluded without data recorded, these patients had no any information recorded at all. There was large non-adherence with the study treatments in our study, and the non-adherence was asymmetric between the groups. A reasonable hypothesis is that physicians administered nalbuphine in the fentanyl group based on the assessment of the potential benefit in the use of nalbuphine as compared with fentanyl. However, the per-protocol analysis did not find a difference between the two groups. Furthermore, the high non-adherence rate could decrease what might have been a significant difference between the two groups. The direction of the bias in the use of other opioids is also difficult to anticipate. Patients in the nalbuphine group received many other opioids; whether the interaction of different opioids strongly inhibits the GI tract and prolongs the defecation time in ICU patients is unclear.

This study has several limitations. First, clinicians were allowed to use opioid analgesics other than study drugs, and we did not exclude the effect of other opioid administrations in the ICU. We had some patients who received drugs that were different from the results of randomization. The cross-use of the study drugs and the large non-adherence may both mask the real difference between nalbuphine and fentanyl (28). We used mITT analysis, not an ITT analysis in our study, which increased the bias. Second, we did not consider the opioid administration during the surgery. Third, we had a short observation time. Fourth, we did not have detailed nutritional information, which is important for maintaining a normal GI function. Fifth, we did not record detailed information about the sedation and the analgesia, although analgesia dose was balanced between the two groups. Sixth, we only chose the first defecation time as the primary outcome ignoring bowel sounds recovery, abdominal pressure, gastric retention, and also some biomarkers, such as the plasma citrulline and plasma or urinary intestinal fatty acid-binding protein, all of these can also provide important information about the GI function in critically ill for our study. Finally, our study was conducted in China, the external validity was limited.

## Conclusion

Our study found no statistically significant difference between nalbuphine and fentanyl regarding the time to first defecation in ICU patients after surgery; however, the *CI* was wide and we could not exclude the clinically important difference.

## Data Availability Statement

The datasets presented in this article are not readily available because the information was not publicly available according to the local law. Requests to access the datasets should be directed to Yu Chen, chenyu007@gmail.com.

## Ethics Statement

The studies involving human participants were reviewed and approved by the Ethics Committee of Xijing Hospital (No. KY20192123-F-1). The patients/participants provided their written informed consent to participate in this study.

## Author Contributions

XZ and YC contributed to the concept and the design of the study. YY drafted the work. YY, ED, GW, BL, XL, AL, and GG performed the subject enrolment, data collection, and research governance. CL, BS, and YY analyzed the data. All authors revised it critically for important intellectual content, read, and approved the final manuscript.

## Funding

This work was supported by the National Natural Science Foundation of China (81871603) and the Academic Abroad Plans of Xijing Hospital (XJZT19Z27 and XJZT19X03).

## Conflict of Interest

The authors declare that the research was conducted in the absence of any commercial or financial relationships that could be construed as a potential conflict of interest.

## Publisher's Note

All claims expressed in this article are solely those of the authors and do not necessarily represent those of their affiliated organizations, or those of the publisher, the editors and the reviewers. Any product that may be evaluated in this article, or claim that may be made by its manufacturer, is not guaranteed or endorsed by the publisher.
