# Supplementary material for: Effects of Nalbuphine on Gastrointestinal Function in Post-Operative Critical Ill Patients Admitted to the ICU: A Multicenter Randomized Controlled Trial
Source: Front Med (Lausanne). 2022 Feb 16;9:836872. doi: 10.3389/fmed.2022.836872 (PMC8888859; doi:10.3389/fmed.2022.836872)
Supplement: Supplementary file 1 [file Data_Sheet_1.docx]

Supplementary Material

**Effects of Nalbuphine on Gastrointestinal Function in Postoperative Critical Ill Patients Admitted to the ICU: a Multicenter Randomized Controlled Trial**

**Index**

[Table S1. The baseline characteristics in the per-protocol population 2](#_Toc88340642)

[Table S2. The baseline characteristics in the as-treated population 3](#_Toc88340643)

[Table S3.The outcomes in the per-protocol population 4](#_Toc88340644)

[Table S4. The outcomes in the as-treated population 5](#_Toc88340645)

[Table S5. Factors of defecation within 48h in the ICU 6](#_Toc88340646)

[Table S6. Recruitment number by site and treatment group in the mITT 7](#_Toc88340647)

[Fig S1. Time to first defecation between the nalbuphine and fentanyl groups in the per-protocol population 8](#_Toc88340648)

[Fig S2. Time to first defecation between the nalbuphine and fentanyl groups in the as-treated population 9](#_Toc88340649)

[Fig S3. The nonadherence of study treatment in the study 10](#_Toc88340650)

# Table S1. The baseline characteristics in the per-protocol population

| **Characteristics** | **Nalbuphine**  **(n=145)** | **Fentanyl**  **(n=112)** |
| --- | --- | --- |
| Age, median[IOR], years | 53.00 [44.00, 62.00] | 51.00 [37.75, 63.25] |
| Sex n(%) |  |  |
| Male | 82 (56.6) | 70 (62.5) |
| Female | 63 (43.4) | 42 (37.5) |
| Body mass index  median [IQR], kg/m2 | 23.66 [21.45, 25.39] | 22.88 [21.38, 25.25] |
| APACHE.II median [IQR] | 8.00 [6.00, 11.00] | 8.00 [5.00, 11.25] |
| Diagnosis on admission |  |  |
| Trauma, n (%) | 84 (57.9) | 59 (52.7) |
| Spinal disease, n (%) | 24 (16.6) | 19 (17.0) |
| Digestive disease, n (%) | 7 ( 4.8) | 10 ( 8.9) |
| Other, n (%) | 30 (20.7) | 24 (21.4) |
| AGI Grade |  |  |
| I, n (%) | 126 (86.9) | 87 (77.7) |
| II, n (%) | 19 (13.1) | 25 (22.3) |
| Lactate median [IQR], mmol/L | 1.60 [1.20, 2.10] | 1.80 [1.10, 2.52] |
| K^+^ median [IQR], mmol/L | 4.00 [3.70, 4.23] | 4.00 [3.70, 4.32] |
| Surgical site |  |  |
| Abdomen, n (%) | 24 (16.6) | 19 (17.0) |
| Limbs, n (%) | 7 ( 4.8) | 10 ( 8.9) |
| Cervical, n (%) | 84 (57.9) | 59 (52.7) |
| Other, n (%) | 30 (20.7) | 24 (21.4) |
| Systolic pressure median [IQR], mmHg | 129.00 [112.00, 142.00] | 125.00 [115.00, 138.25] |
| Diastolic pressure median [IQR], mmHg | 75.00 [66.00, 83.00] | 75.00 [63.75, 81.25] |

# Table S2. The baseline characteristics in the as-treated population

| **Characteristics** | **Nalbuphine**  **(n=203)** | **Fentanyl**  **(n=130)** |
| --- | --- | --- |
| Age, median[IOR], years | 53.00 [41.00, 64.00] | 53.00 [41.00, 64.00] |
| Sex n(%) |  |  |
| Male | 112 (55.2) | 81 (62.3) |
| Female | 91 (44.8) | 49 (37.7) |
| Body mass index median [IQR],kg/m2 | 23.81 [22.04, 25.39] | 23.05 [21.37, 24.75] |
| APACHE.II median [IQR] | 9.00 [6.00, 12.00] | 8.00 [5.00, 12.00] |
| Diagnosis on admission |  |  |
| Trauma, n (%) | 110 (54.2) | 67 (51.5) |
| Spinal disease, n (%) | 31 (15.3) | 21 (16.2) |
| Digestive disease, n (%) | 110 (54.2) | 67 (51.5) |
| Other, n (%) | 52 (25.6) | 30 (23.1) |
| AGI Grade |  |  |
| I, n (%) | 177 (87.2) | 107 (82.3) |
| II, n (%) | 26 (12.8) | 23 (17.7) |
| Lactate median [IQR], mmol/L | 1.60 [1.20, 2.10] | 1.60 [1.10, 2.48] |
| K^+^ median [IQR], mmol/L | 4.00 [3.70, 4.28] | 4.00 [3.70, 4.30] |
| Surgical site |  |  |
| Abdomen, n (%) | 32 (15.8) | 26 (20.0) |
| Limbs, n (%) | 59 (29.1) | 31 (23.8) |
| Cervical, n (%) | 58 (28.6) | 37 (28.5) |
| Other, n (%) | 54 (26.6) | 36 (27.7) |
| Systolic pressure median [IQR], mmHg | 127.67 [112.00, 140.50] | 123.50 [114.00, 137.25] |
| Diastolic pressure median [IQR], mmHg | 75.00 [65.00, 81.00] | 75.00 [63.00, 81.00] |

# Table S3.The outcomes in the per-protocol population

| **Outcomes** | **Nalbuphine**  **(n=145)** | **Fentanyl**  **(n=112)** | **P value** | **RR 95%CI** |
| --- | --- | --- | --- | --- |
| AGI Grade |  |  | 1 | 0.95(0.48-1.87) |
| I, n (%) | 122 (84.1) | 95 (84.8) |  | 0.97(0.73-1.31) |
| II, n (%) | 23 (15.9) | 17 (15.2) |  | 1.03(0.69-1.52) |
| Vomit, n (%) | 23 (15.9) | 20 (17.9) | 0.798 | 0.93(0.69-1.27) |
| Sedation^a^ |  |  | 0.386 |  |
| Propofol, n (%) | 13 ( 9.0) | 16 (14.3) |  |  |
| Dexmedetomidine, n (%) | 97 (66.9) | 64 (57.1) |  |  |
| Midazolam, n (%) | 7 ( 4.8) | 6 ( 5.4) |  |  |
| Enema/Laxative, n (%) | 37 (25.5) | 25 (22.3) | 0.655 | 1.07(0.84-1.37) |
| Length of ICU stay, median [IQR], days | 3.00 [2.00, 5.00] | 3.00 [2.00, 4.25] | 0.393 | 0 |
| Hospital stay, median [IQR], days | 11.00 [8.00, 16.00] | 11.00 [7.00, 16.00] | 0.361 | 1(-1-2) |

# Table S4. The outcomes in the as-treated population

| **Outcomes** | **Nalbuphine**  **(n=203)** | **Fentanyl**  **(n=130)** | **P value** | **RR 95%CI** |
| --- | --- | --- | --- | --- |
| AGI Grade |  |  |  | 0.85 (0.46-1.60) |
| I, n (%) | 171 (84.2) | 112 (86.2) | 0.749 | 0.94 (0.75-1.18) |
| II, n (%) | 32 (15.8) | 18 (13.8) |  | 1.09 (0.73-1.63) |
| Vomit, n (%) | 30 (14.8) | 23 (17.7) | 0.579 | 0.91 (0.81-1.59) |
| Sedation^a^ |  |  | 0.51 |  |
| Propofol, n (%) | 15 ( 7.4) | 16 (12.3) |  |  |
| Dexmedetomidine, n (%) | 124 (61.1) | 76 (58.5) |  |  |
| Midazolam, n (%) | 11 ( 5.4) | 7 ( 5.4) |  |  |
| Enema/Laxative, n (%) | 54 (26.6) | 30 (23.1) | 0.951 | 1.07 (0.89-1.29) |
| Length of ICU stay, median [IQR], days | 3.00 [2.00, 4.00] | 2.00 [2.00, 4.00] | 0.564 | 0 |
| Hospital stay, median [IQR], days | 12.00 [8.00, 17.00] | 11.00 [7.00, 16.00] | 0.143 | 1 (0-2) |

# Table S5. Factors of long defecation within 48h in the ICU

| **Variables** | **Univariate** | | **Multivariable** | |
| --- | --- | --- | --- | --- |
|  | **OR (95%CI)** | **P value** | **OR (95%CI)** | **P value** |
| Female | 0.68(0.44-1.04) | 0.08 |  |  |
| Surgical site |  |  |  |  |
| Limbs | 0.51(0.27-0.97) | 0.04 | 0.44(0.22-0.87) | 0.02 |
| Cervical | 0.99(0.53-1.85) | 0.98 |  |  |
| Others | 0.56(0.29-1.07) | 0.08 |  |  |
| APACHE II | 1.05(1.01-1.1) | 0.03 | 1.06(1.01-1.12) | 0.01 |
| Sedation | 1.96(1.21-3.17) | 0.01 | 2.2(1.31-3.71) | <0.01 |
| Enteral nutrition ≤ 48h | 1.1(0.67-1.8) | 0.71 |  |  |
| Laxative/Enema | 0.7(0.44-1.13) | 0.14 |  |  |
| Vasoactive | 1.76(0.95-3.27) | 0.07 |  |  |
| Mechanical ventilation | 1.2(0.7-2.07) | 0.5 |  |  |

# Table S6. Recruitment number by site and treatment group in the mITT

| Site | Number of patients in nalbuphine group | Number of patients in fentanyl group | Total |
| --- | --- | --- | --- |
| 1 | 110 | 100 | 210 |
| 2 | 51 | 41 | 92 |
| 3 | 9 | 25 | 34 |
| 4 | 1 | 2 | 3 |
| 5 | 2 | 2 | 4 |
| 6 | 3 | 9 | 12 |
| 7 | 9 | 5 | 14 |
| Total | 185 | 184 | 369 |

Site1, Xijing hospital; Site2, the Affiliated Honghui Hospital of Xi'an Juwtong University; Site3, 521 Hospital of NORINCO GROUP; Site4, Qinghai University Affiliated Hospital; Site5, Aerospace General Hospital; Site6, The Second Affiliated Hospital of Xi'an Medical University; Site7, The First Hospital of Lanzhou University

# Fig S1. Time to first defecation between the nalbuphine and fentanyl groups in the per-protocol population

# Fig S2. Time to first defecation between the nalbuphine and fentanyl groups in the as-treated population

# Fig S3. The nonadherence of study treatment in the study
